# Identification of Navβ1 Residues Involved in the Modulation of the Sodium Channel Nav1.4

**DOI:** 10.1371/journal.pone.0081995

**Published:** 2013-12-16

**Authors:** Angel A. Islas, Alfredo Sánchez-Solano, Thomas Scior, Lourdes Millan-PerezPeña, Eduardo M. Salinas-Stefanon

**Affiliations:** 1 Laboratorio de Biofísica, Instituto de Fisiología, Universidad Autónoma de Puebla, Puebla, Mexico; 2 Facultad de Ciencias Químicas, Universidad Autónoma de Puebla, Puebla, Mexico; 3 Centro de Química, Instituto de Ciencias, Universidad Autónoma de Puebla, Puebla, Mexico; University of Pittsburgh School of Medicine, United States of America

## Abstract

Voltage-gated sodium channels (VGSCs) are heteromeric protein complexes that initiate action potentials in excitable cells. The voltage-gated sodium channel accessory subunit, Navβ1, allosterically modulates the α subunit pore structure upon binding. To date, the molecular determinants of the interface remain unknown. We made use of sequence, knowledge and structure-based methods to identify residues critical to the association of the α and β1 Nav1.4 subunits. The Navβ1 point mutant C43A disrupted the modulation of voltage dependence of activation and inactivation and delayed the peak current decay, the recovery from inactivation, and induced a use-dependent decay upon depolarisation at 1 Hz. The Navβ1 mutant R89A selectively delayed channel inactivation and recovery from inactivation and had no effect on voltage dependence or repetitive depolarisations. Navβ1 mutants Y32A and G33M selectively modified the half voltage of inactivation without altering the kinetics. Despite low sequence identity, highly conserved structural elements were identified. Our models were consistent with published data and may help relate pathologies associated with VGSCs to the Navβ1 subunit.

## Introduction

Voltage-gated sodium channels (VGSCs) are responsible for eliciting action potentials in excitable cells [1]. Abnormal activity and expression of VGSCs have been associated with cardiac and neuropathic pathologies including epilepsy, inflammatory processes, and periodic paralysis [2]. Mammals possess 11 genes clustered on 4 chromosomes, which produce 9 functional VGSCs, Nav1.1–Nav1.9 [3].

Sodium channels are multi heteromeric integral proteins composed of one pore-forming α subunit that associates with one or more auxiliary β subunit. The α subunit (260 kDa) contains 4 homologous domains (I–IV), and each domain contains 6 membrane-spanning sequences (S1–S6). These 4 domains form the voltage-dependent, cation-selective sodium channel [4]. Extracellular and intracellular loops of variable sizes connect the 6 transmembrane helices of the 4 domains [4].

The β subunits are single-pass membrane glycoproteins (30–40 kDa) with large N-terminal immunoglobulin like (Ig-like) extracellular domains and smaller C-terminal intracellular domains [5]. Four distinct isoforms (β1–β4) have been identified. They act as multifunctional cell adhesion molecules (CAMs) and interact with extracellular matrix proteins and cytoskeletal and cytosolic signalling proteins [2].

The β1 subunit modulates the voltage dependence and kinetics of the pore-forming α subunit and can increase inactivation rates and promote faster recovery from inactivation in an isoform and cell-type specific manner. Previous studies have indicated that non-covalent extracellular interactions between α and β1 subunits mediate the functional modulation [6], [7], [8], [9], [10], [11], [12], [13], however the molecular nature of the interactions remain poorly understood [2].

The Myelin protein P0 has been used to model the association of the α and β1 subunits in potassium channels [14]. Using multiple sequence alignments (MSAs) and evolutionary information, conserved amino acids can be identified. Often, these sites of conservation are important for the structure or function of proteins. In addition, family-dependent conserved positions that differ in the chemical type of amino acids can dictate binding to other proteins [15]. Studies that combine computational predictive methods, including MSA, knowledge-based evolutionary information, and secondary structure predictions yield an estimated 85% success rate for predicting amino acids involved in protein-protein associations [16].

Early studies from our laboratory, using oocytes co-injected with β1–β2 chimeras, were consistent with previous findings that indicate the extracellular domain of β1 accounts for the regulation of Nav1.4 channels [13], [17]. This β1 domain belongs to the antibody variable domain family of Ig-like proteins [12]. In the present study, we combine electrophysiology, site-directed mutagenesis, and *in silico* methods to investigate the structure-function relationships of the VGSC β1 on the Nav1.4 α isoform. To assess the importance of a putative intramolecular disulphide bridge and a salt bridge in Navβ1 subunit we generated alanine substitutions C43A and R89A that would disrupt these bonds. According to our model, the turn of a loop on β1, may also be important in the modulation of the sodium channel. To test this hypothesis we neutralize the aromatic side chain of Y32 and introduce a bulky side chain in G33 at this loop turn with the mutants Y32A and G33M, respectively and tested their effects on Nav1.4 channels.

## Materials and Methods

### Site-directed Mutagenesis and Heterologous Expression

The rNav1.4 cDNA cloned into the pGW1H vector was kindly provided by Eduardo Marban (John Hopkins University). Several point mutants were generated on the rNavβ1 construct (Scnb1: Q00954) using the QuikChange® II XL site-directed mutagenesis kit (Stratagene, La Jolla, CA, USA). The integrity of the PCR products were verified by electrophoresis and transformed by heat shock into ultracompetent One Shot® bacteria for amplification and nick repair. After antibiotic selection and culture, the mutated plasmids were obtained using the HiSpeed® Plasmid Purification kit (Qiagen, Mexico city, Mexico). Successful mutagenesis was confirmed by DNA sequencing with the Applied Biosystems 3730 DNA Analyzer (UNAM, Cuernavaca, Mexico).

Frog oocytes were chosen for heterologous expression because some mammalian cell lines such as HEK-293 express endogenous β1 splice variants [18]. Adult *Xenopus laevis* female frogs (Xenopus 1, Dexter, MI, USA) were anesthetized by immersion in 0.2% tricaine (Sigma Chemical, St Louis MO, USA). Oocytes were surgically removed, placed in OR-2 buffer containing (in mM) 82.5 NaCl, 2.5 KCl, 1 MgCl_2_, and 5 4-2-hydroxyethylpi-97perazine-1-ethanesulfonic acid (HEPES), pH 7.6, then treated with collagenase 1.3 mg/mL to remove the follicular membrane. The nuclei of stage V and VI oocytes were injected using a nanolitre automatic injector (model A203XVY, WPI, Sarasota, FL, USA) with 25 to 30 ng of rNav1.4 cDNA in a 1∶5 ratio of WT or mutant β1. Eggs were then maintained for up to 3 days at 18°C in ND-96 solution (in mM), 96 NaCl, 2 KCl, 1 MgCl_2_, 5 HEPES, and 1 CaCl_2_, supplemented with 0.5 mM theophylline, 0.5 mM pyruvate, and 50 µg/mL gentamicin. The pH was adjusted to 7.6 with 1 M NaOH. Oocytes in recording chambers were continuously superfused at a flow rate of 500 µL/min with ND-96 solution with 1 mM BaCl_2_ without CaCl_2_.

All surgical procedures were performed in accordance with the Guide for the Care and Use of Laboratory Animals from the Mexican Council for Animal Care (Norma Oficial Mexicana NOM-062-ZOO-1999) and the National Institutes of Health Guide for the Care and Use of Laboratory Animals. This study was approved by the internal ethics committee of the Instituto de Fisología of the Universidad Autónoma de Puebla. All efforts were made to reduce the number of animals used and minimize animal suffering.

### Electrophysiological Recording and Data Analysis

Two electrode voltage-clamp recordings were performed at room temperature (20–22°C) using an OC-725 C amplifier (Warner, New Haven, CT). The electrodes were pulled on a horizontal P-97 puller (Sutter Instruments, Novato, CA) and filled with 3 M KCl with a resistance of 0.6 to 1.2 MΩ. The sodium-current signals were filtered at 1 kHz, digitized at a sampling rate of 10 kHz by a Digidata 1200 analogue-to-digital converter (Axon Instruments, Foster City, CA), and stored on a computer for analysis with pClamp software Version 8.02 (Axon Instruments, Foster City, CA). The sodium currents (I_Na_) were generated by step depolarisations from a holding potential of −100 mV at 0.1 Hz, unless otherwise indicated. To minimize voltage-clamp errors, only oocytes with minimal leak (≤0.1 µA) were used in the study. Capacitive currents were manually compensated. Sodium conductances (gNa) were obtained from the peak currents generated by step depolarisations of 10 mV increments (30 ms durations) from a holding potential of −100 mV to 0 mV and calculated with equation:
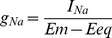
where *Em* was the membrane potential, *I_Na_* was the current amplitude, and *E_eq_* was the equilibrium potential calculated for each cell. Steady-state inactivation data were obtained using a two-pulse protocol. First, a variable voltage conditioning pulse (from −120 mV to 0 mV, 1000 ms duration) was given from a holding potential of −100 mV and a gap of 2 ms. Second, a test pulse (25 ms duration) at −20 mV was given. The peak current from the second pulse was plotted as a function of the conditioning pulse potential. Activation and steady-state inactivation curves from every group were fitted using the Boltzmann distribution equation:



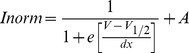
where *V* was the potential of the voltage pre-pulse, *V*_1/2_ was the half voltage of inactivation, *dx* was the slope, and *A* was a residual linear component. The time course of inactivation data from the peak current at −20 mV was fitted to a single exponential equation:



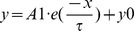
where *A1* was the relative fraction of current inactivation, *τ* was the time constant, and *x* was the time. The recovery from inactivation was examined using a 500 ms conditioning pulse at −20 mV from a holding potential of −100 mV followed by a variable recovery interval (Δt = 1 to 10000 ms) and a test pulse at −20 mV. Recovery data from each cell were fitted by a double exponential equation:



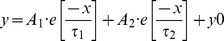
where *A_1_* and *A_2_* were relative fractions of the recovery currents, *τ1* and *τ2* were time constants, and *y0* was the amplitude of the steady-state component. The decay current after repetitive stimulation was determined by applying trains of 30 ms pulses from a holding potential of −100 mV to a test potential of −20 mV at 1 Hz. Current amplitudes were normalized with respect to the first pulse. All the currents were analyzed using the pClamp version 10.2 software (Axon Instruments, Foster City, CA). Values were shown as mean ± SEM. Statistical comparisons between mutant and wild type mean values were performed using unpaired Student’s t-test. Comparisons of more than two mean values were performed using one-way analysis of variance (ANOVA), and pairwise multiple comparisons were performed using the Holm-Sidak method. The graphs were built and fitted using Sigmaplot 11.0 (SPSS, Inc., Chicago, Il) and Origin 8.02 (OriginLab Corp., Northampton, MA, USA).

### Homology and Molecular Modeling

Various atomic models of rNavβ1 were generated including fully and partially unattended homology models using three approaches: (1) I-TASSER [19], (2) Geno3D [20] and (3) CPHmodels-3.0 [21]. Model refinements were performed under the AMBER Force Field (FF) in VEGA ZZ 2.3.2 [22] and the UCSF Chimera [23]. The stereochemical qualities of the models were assessed with the VADAR server [24]. For model comparisons, sequential hydropathy analyses were performed using the MPex 3.2 software [25], and secondary structure predictions were performed using the web-based Jpred3 [26]. An inter-species MSA of β1 was performed to identify highly conserved domains involved in VGSC modulation. Sequence alignments were obtained using several algorithms and best scores were obtained using Multiple Sequence Comparisons by Log-Expectation MUSCLE [27]. Three-dimensional Smith-Waterman alignments using the BLOSUM-62 matrix with weighted secondary structure similarities (30%) were used to superpose structures [28]. Electrostatic surface potential (ESP) was calculated with Chimera under AMBER FF and coloured with the Coulombic scale for display with equation:
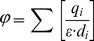
where *φ* was the potential, *q* was the atomic partial charges, and *d* was the distance from the atoms *i.* The dielectric constant *ε = *4 was used to represent screening by the medium or solvent. For the purpose of this study the residue numbers included the signal peptide. However, this region is cleaved to form the quaternary structure of the membrane spanning protein, and it has been excluded from models and simulations.

## Results

Several homology models of β1 were generated using the crystallized myelin protein P0 as the template, which had the highest degree of homology to β1. This protein associates with the VGSC subunits without affecting its electrophysiological behaviour [10]. The rat sequence of myelin protein P0 (PDB code: 1neu) was 25% identical to the extracellular Ig-like domain of rβ1 with 63% positive substitutions of 113 paired target residues comprising 80% of the entire extracellular domain.

**Figure 1:**
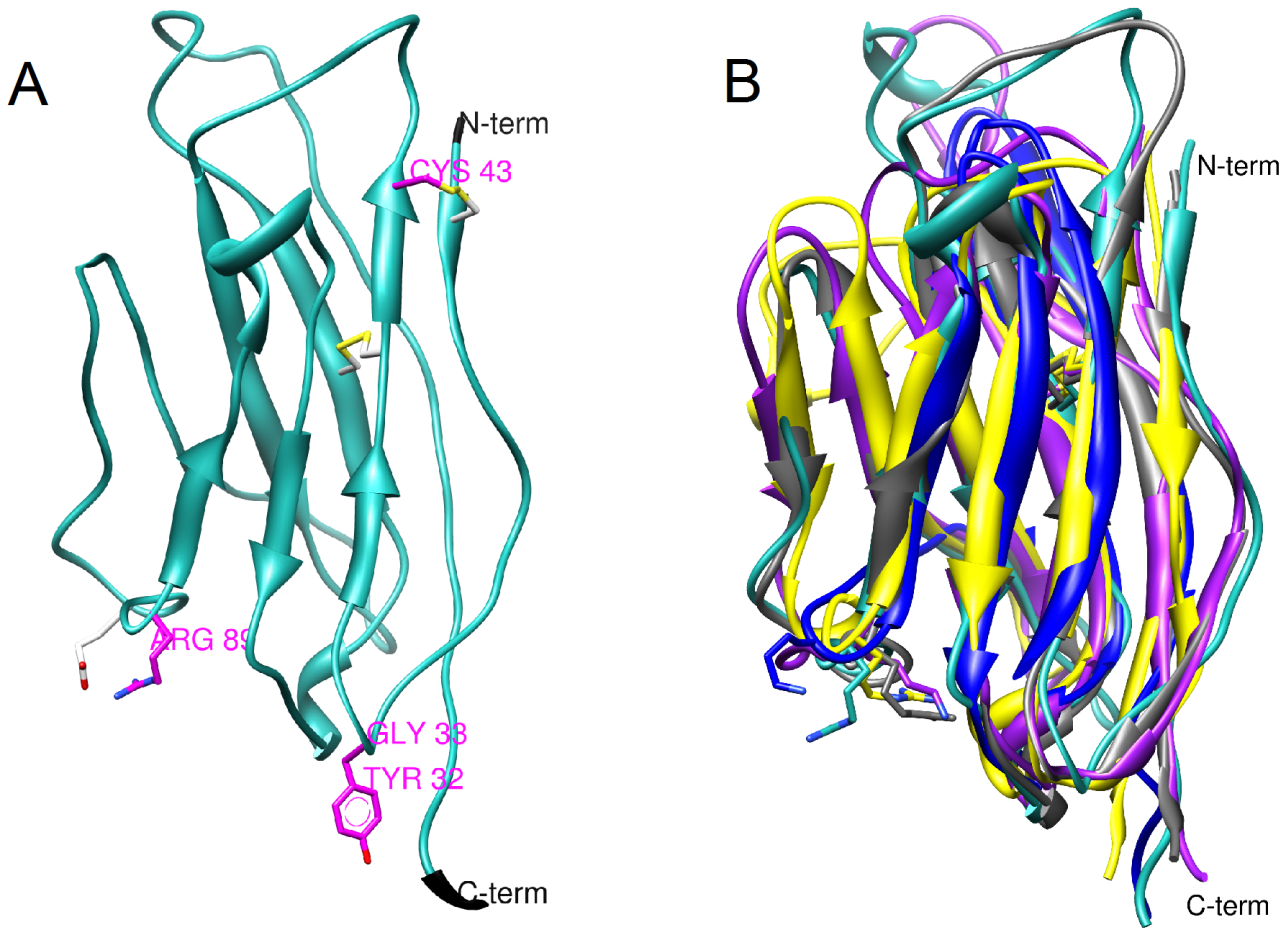
Atomic homology model of the extracellular domain of the Navβ1 subunit. (A) The functionally relevant residues identified are shown in magenta. Cysteine 43 was predicted to form a disulphide bond with cysteine 21 and arginine 89 may form a salt bridge with glutamate 87. The buried disulphide bridge between C40 and C121 is also shown. (B) The model of β1 (cyan) was superimposed over the template structure, myelin protein P0 (gray), and 3 Ig-like proteins including NTRK2_HUMAN, TRGC2_HUMAN, and a camelid VHH antibody (PDB codes: 1neu_A, 1wwb_X, 1hxm_B, 1kxq_H). Despite their divergent functions, the general topologies of the crystal structures were conserved. The conserved basic residues at the position equivalent to Arg89 are displayed as atom sticks (colour code: β1 model in cyan, 1wwb_X in marine blue, 1hxm_B in magenta, and 1kxq_H in yellow).

Figure 1A shows the structural model of the extracellular domain of the sodium channel β1 subunit, and figure 1 B shows this three-dimensional representation of Navβ1 (in cyan) superposed to its template (in gray) and 3 Ig-like crystal structures including a Tyrosine kinase, a T-cell receptor, and an antibody (PDB codes: 1wwb_X, 1hxm_B, 1kxq_H, respectively). Based on structure, knowledge, and interspecies MSAs (Figure S1), 2 sites that may form intramolecular bonds critical to the modulation of the sodium channel were identified. First, cysteine 43 is conserved across species in β1 and the model of the tertiary structure of β1 indicated that it lies close to C21, a cysteine near the N-terminus. Previous data indicates the cysteine corresponding to C43 forms a disulphide bond in β3 [29]. Second, highly conserved arginine 89 was proposed to form an exposed intramolecular salt bridge with an adjacent acidic residue. To assess the importance of these residues, we generated alanine substitutions C43A and R89A and tested their effects on Nav1.4 channels.

Our modelling results indicated that Arg89 formed a salt bridge with Glu87 (<2.5 Å distance). Alternatively, it may interact with the carbonyl main chain of Val111. The equivalent position of R89 on the template (Arg69) also formed a salt bridge.

It has been acknowledged that low homology crystal structures conserve relevant three-dimensional information [30], which may be consistent with this case. The superposition of other Ig-like crystal structures indicated the potential to form an electrostatic bond at this position was conserved through divergent evolution. The residue at position 89 in β1 was always a basic residue (arginine or lysine, Figure 1B stick atom representation) despite very low sequence identity (<25%). In particular, two proteins showed salt bridges between arginine and aspartate or glutamate within a range of 5 Å. Finally, the Smith-Waterman 3D alignment yielded score values of 25%, 16%, and 28% homology for the three Ig-like crystal structures.

**Figure 2:**
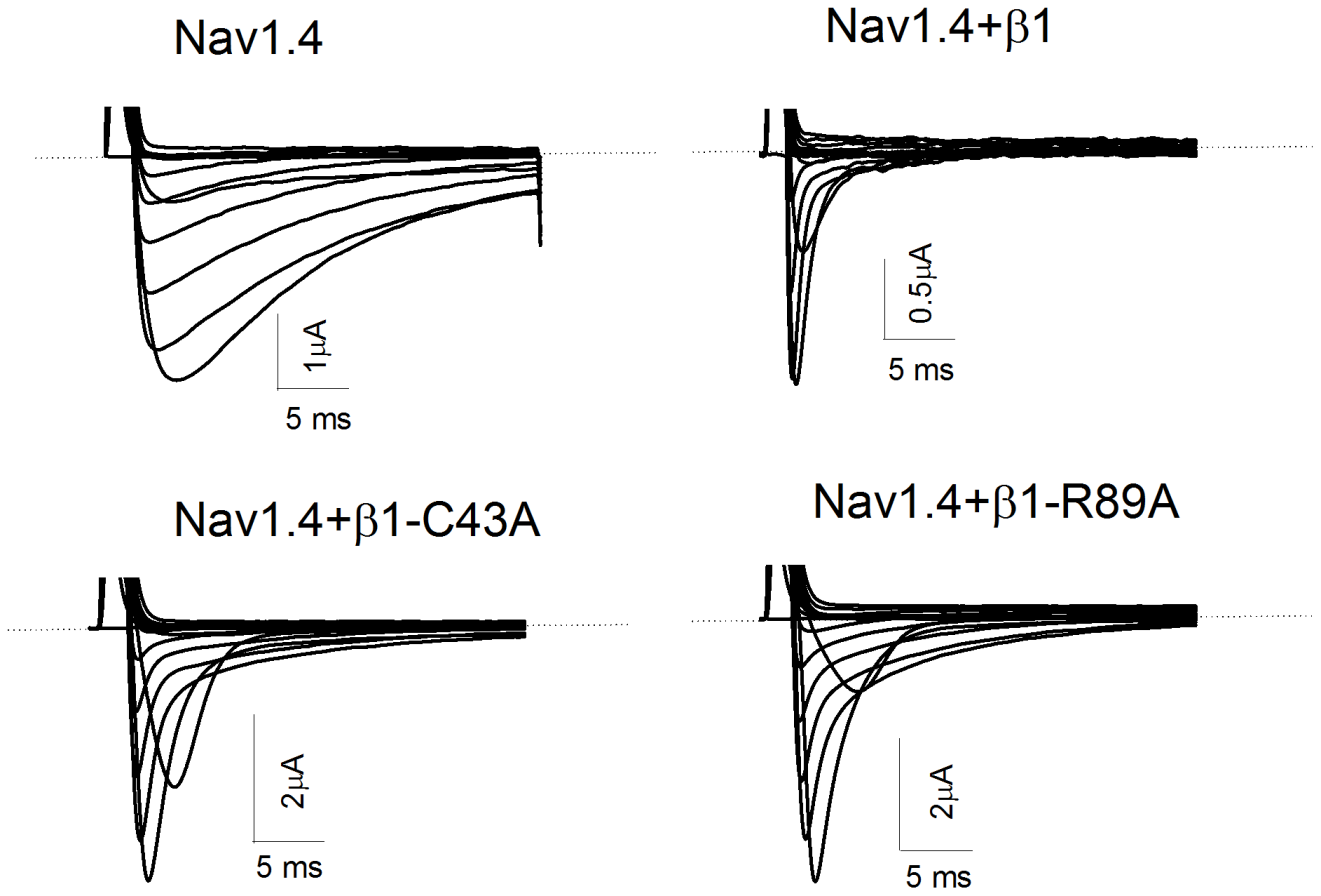
Representative current traces of Nav1.4 channel in the absence and presence of β1 and point mutants. Sodium currents were generated by step depolarisations from a holding potential of −100 mV in 10 mV increments from −100 mV to +50 mV (30 ms duration).

Wild type (WT) β1 accelerated the inactivation and the recovery from inactivation, and shifted the voltage dependence of activation and inactivation to more hyperpolarized potentials. Figure 2 showed representative current traces for the Nav1.4 channel stably co-expressed on frog oocytes alone and with the β1 subunit and the point mutants C43A and R89A. The effects on the current decay were disrupted by the alanine substitutions at Cys43 and Arg89 (Figure 2).

### β1-C43A Disrupts Modulation of the Voltage Dependence on Nav1.4 Channels

**Figure 3:**
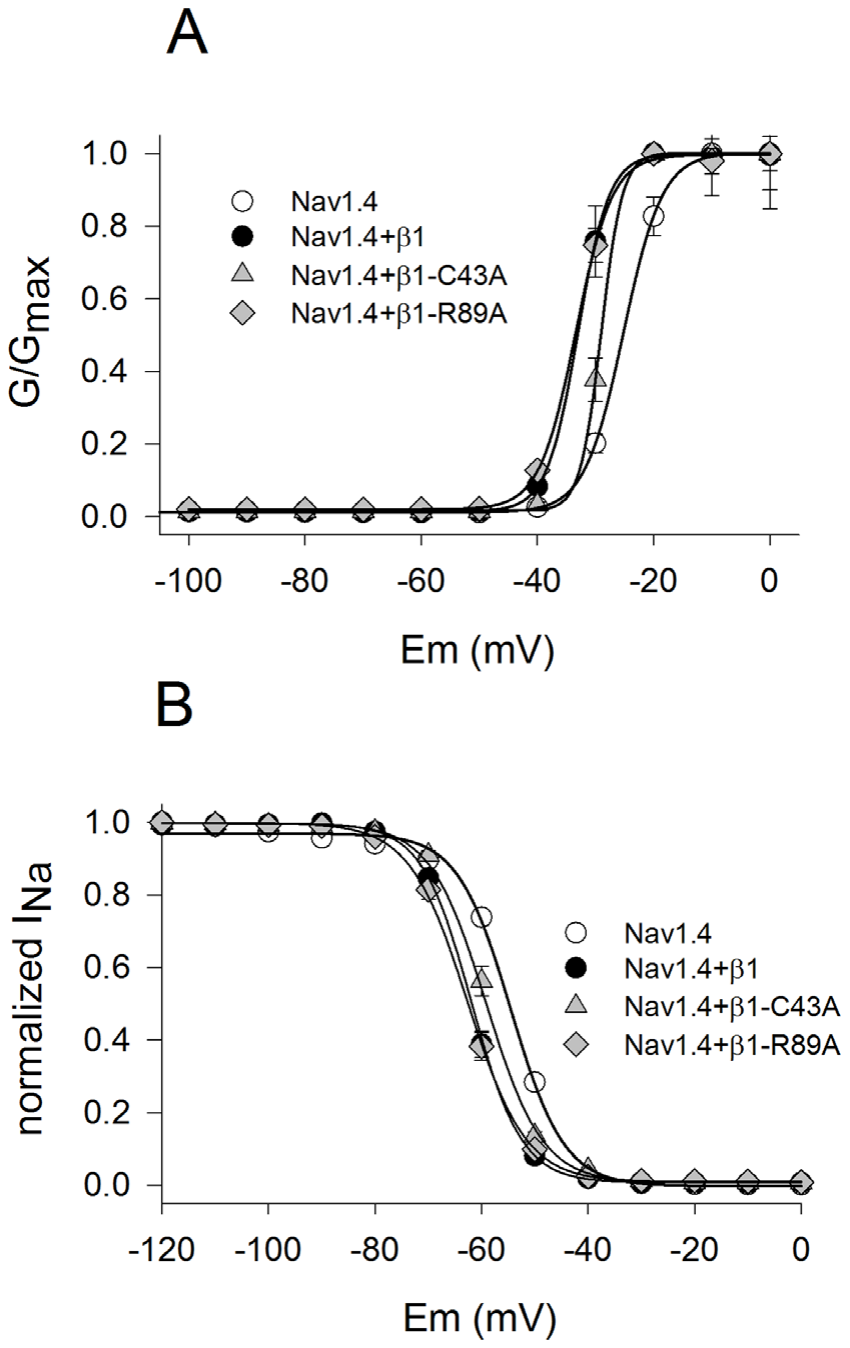
Effects of β1 mutants on Nav1.4 channel voltage-dependence of activation and inactivation. (A) Normalized conductance plotted as a function of the voltage potential of Nav1.4 channels expressed alone and with β1 and each mutant (inset). (B) Steady-state inactivation curves of the same groups. Data were expressed as mean ± SEM (n = 5–9). Data were fitted with a Boltzman equation (protocols described in methods).

Figure 3 shows the hyperpolarizing shifts that β1 induced on activation and steady-state inactivation on the Nav1.4 channel. This modulation was disrupted by the C43A mutant but not by the R89A mutant. The half voltage of activation and inactivation for C43A was significantly different from WT β1 (Table 1) constituting a loss-of-function effect on the voltage dependency.

**Table 1:** Biophysical parameters for voltage-dependence of activation and inactivation.

Channel type	Activation V_1/2_ (mV)	Slope	Inactivation V_1/2_ (mV)	Slope
Nav1.4	−25.9±0.5***	1.8±0.3***	−54.5±0.4***	5±0.31
Nav1.4+β1	−33.4±0.2	3.1±0.1	−61.8±0.3	4.7±0.1
Nav1.4+β1-C43A	−29±0.1***	1.8±0.3***	−59.1±0.4***	6.5±0.2***
Nav1.4+β1-R89A	−33±0.1	2.6±0.1	−62.6±0.5	5.5±0.2
Nav1.4+β1-Y32A	−34±0.1	2.1±0.1**	−56±0.3***	4.6±0.1
Nav1.4+β1-G33M	−32.5±1	2.3±0.1	−56±0.4***	4.7±0.1

= 5–9, *P<0.05, **P<0.01, ***P<0.001). Values were obtained from mean data fitted with the Boltzmann equation. Statistically significant differences were determined with a one-way ANOVA test followed by a Holm-Sidak test for multiple comparisons (n

### β1-C43A and β1-R89A Delayed the Inactivation and Recovery of Nav1.4 Channels

**Figure 4:**
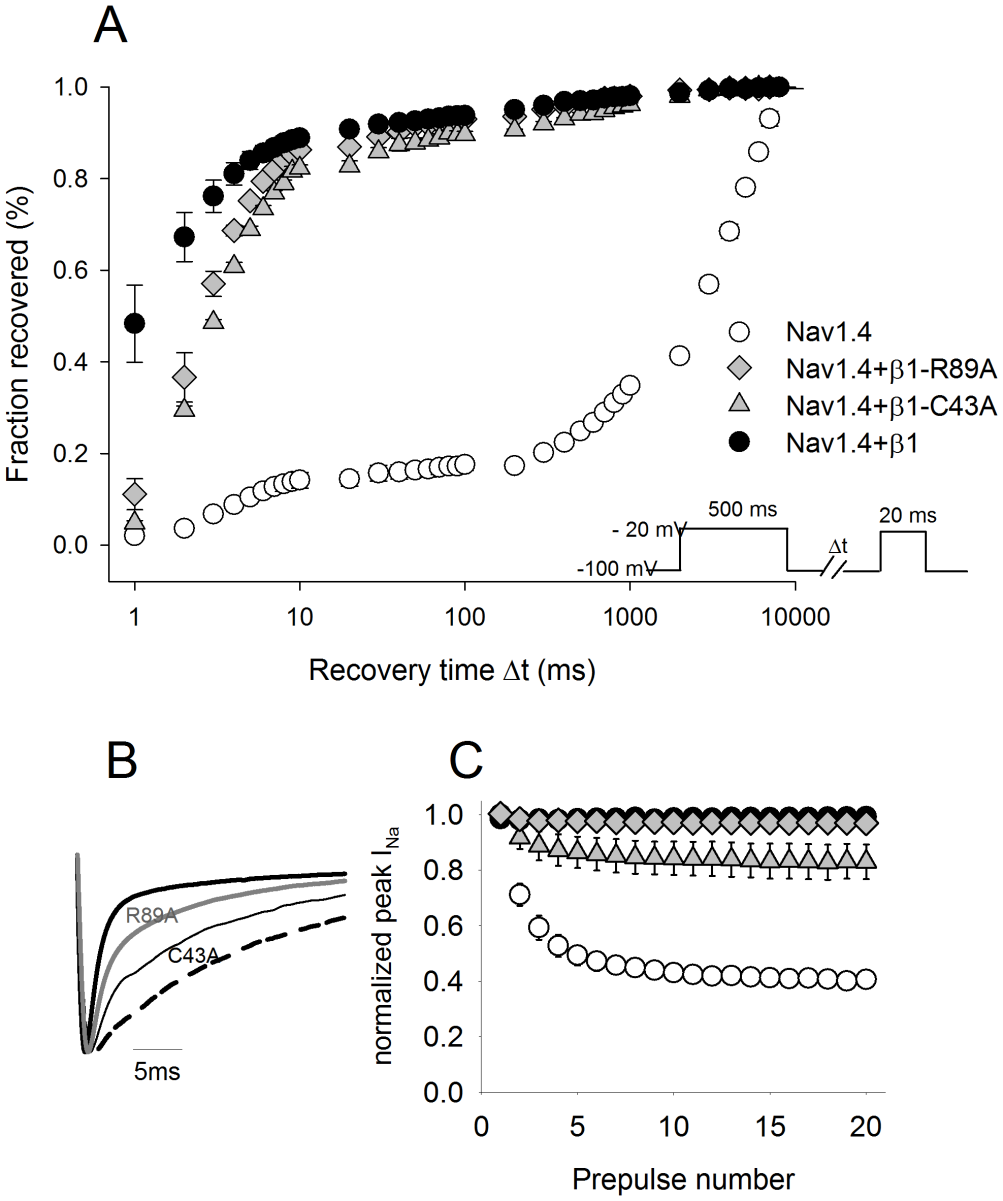
Effects of β1 and mutants on Nav1.4 channel recovery from inactivation. (A) The current amplitude at the second pulse was normalized to the current amplitude at the pre-pulse and plotted as a function of the variable time interval (Δt), currents were evoked according to the protocol in the inset. (B) Representative traces of currents evoked by a depolarizing step of −20 mV (peak current activation). The trace in bold was obtained from WT channels (Nav1.4+β1) and the dashed trace represented the Nav1.4 α channel alone. (C) Effects of β1 and mutants on Nav1.4 after repetitive depolarisations (30 ms duration) from a holding potential of −100 mV to −20 mV at a frequency of 1 Hz. Data were expressed as mean ± SE (n = 4 to 9 cells).

In Figure 4 A, we illustrate the recovery from inactivation. WT β1 generated a current recovery of approximately 50% after 1 ms. R89A and C43A slowed this process by 90% y 95% respectively (*P*<0.05). The recovered fraction of R89A was different from WT after a Δt of 10 ms (*P = *0.05), however C43A was significantly different until a Δt of 100 ms (*P*<0.05). Data from each oocyte from each group was fitted individually and the means of the time constants were compared. Only the time constant of recovery from fast inactivation (τ_fast_) of C43A differed from Nav1.4+β1 (Table 2).

**Table 2:** Parameters for kinetics of inactivation and recovery from inactivation.

Channel type	Inactivation rate τ_inac_ (ms)	Recovery τ_fast_ (ms)	F_fast_	Recovery τ_slow_ (ms)
Nav1.4	NA	4.7±0.4***	0.01	10931±2704***
Nav1.4+β1	0.6±0.03	1.7±0.3	0.93	236±88
Nav1.4+β1-C43A	NA	2.7±0.04**	0.89	636±36
Nav1.4+β1-R89A	2.6±0.3***	2.3±0.1	0.92	376±40
Nav1.4+β1-Y32A	0.48±0.04	NA	NA	743±44
Nav1.4+β1-G33M	0.46±0.03	NA	NA	760±30

+β1 wild type, *s*tatistically significant differences were determined with a one-way ANOVA test followed by a Holm-Sidak test for multiple comparisons (n = 4–9, *P<0.05, **P<0.01, ***P<0.001). The time constants of inactivation were obtained by fitting with single exponential equations, and the time constants of recovery were obtained from fitting with double exponential equations. Each cell was fitted individually and the mean time constants from each group were compared with the Nav1.4

We analyzed the effects of the mutants on current decay and found that although both mutants decrease the β1-induced acceleration of inactivation (Figure 4 B), R89A yielded a statistically significant inactivation time constant, and C43A induced an irregular decay that did not fit an exponential equation. This behaviour was similar to the behaviour of the Nav1.4 α subunit alone (Table 2).

### β1-C43A Induced a Use-dependent Decay

The presence of β1 also allows some isoforms of the sodium channel to recover from high frequency depolarisations. As shown in Figure 4 C, the current from the α subunit of Nav1.4 alone declined 30% after the second pulse at 1 Hz and exhibited a cumulative rundown up to 60% from control after repetitive peak voltage stimulations. Mutant C43A induced a 10% decline after the second pulse and had a cumulative rundown of 20% from control as opposed to R89A and WT β1. At 5 Hz, we observed the same 20% rundown for the C43A mutant and no effects for WT β1 or the R89A mutant (data not shown).

### Computed Physicochemical Effects of β1 Mutants

**Figure 5:**
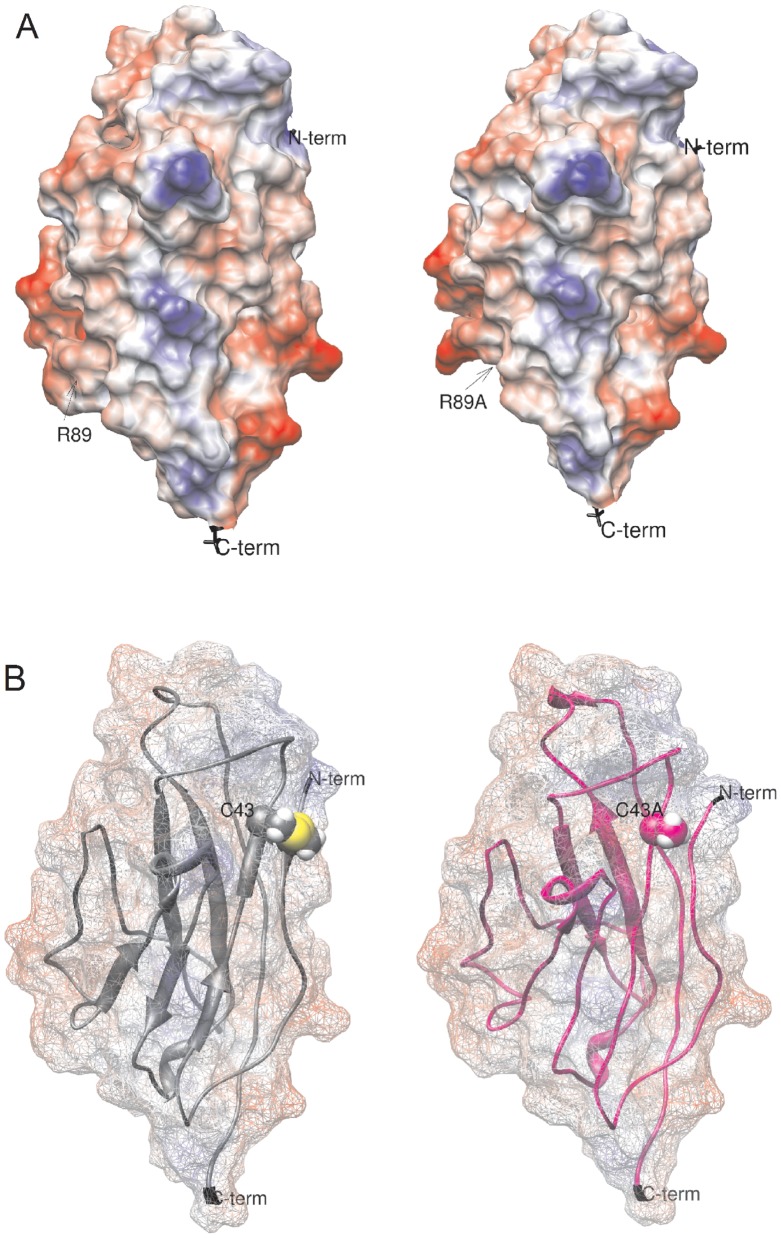
Effects of β1 single residue substitutions on the electrostatic surface potential (ESP). (A) ESP of the extracellular domain of WT β1 (left) and the R89A mutant (right). The point mutation increased the protein’s negative charge by the loss of a salt bridge between Arg89 and Glu87. The acidic carboxylic side chain was now exposed and reactive. (B) ESP of WT (left) and the C43A mutant (right). This substitution prevented the formation of an intermolecular disulphide bond between C43 and C21, increasing entropy. Calculations were made under the AMBER-Gasteiger force field in Chimera 1.3.5 after energy minimization under AMMP in VEGA ZZ 2.3.2.

To investigate the changes in potential energy produced by each mutant, we calculated the ESP of β1. In Figure 5A, the local increase in acidity produced by the neutralizing substitution of Arg89 for alanine and the resultant loss of the proposed intramolecular salt bridge with Glu87 were shown. Importantly, these electrostatic changes did not affect the voltage dependence of either activation or inactivation. Our model predicted that the C43A mutant produced a local increase in polarity caused by the release of the thiol group of C21 (Figure 5 B, sphere atom representation), however the net effect of this mutant reflected a greater increase in entropy and reduction of rigidity at the N-terminal loop, which seemed to be a critical participant in the modulation of voltage dependence on channel activation and inactivation.

To further evaluate the role of this loop in the β1-induced modulation of the channel, we designed two point mutants in the first turn in the C-terminus direction. We neutralized the aromatic polar side chain of Y32 by an alanine substitution (Y32A), and tested the introduction of a large hydrophobic side chain with the mutant G33M. Each single point mutation selectively disrupted the voltage dependence of inactivation. Y32A and G33M significantly shifted the half voltages of inactivation to more depolarized potentials compared with WT (Table 1). Importantly these mutants did not modify the voltage dependence of activation, the time constants of inactivation, or the recovery from inactivation (Table 1 and 2). Our model showed that the alanine substitution of the Tyr43 aromatic ring uncovered a nearby acidic residue (Asp148) involved in a hydrogen bond network with the Tyr43 hydroxyl group. The substitution of the next residue by methionine (G33M) introduced a bulky hydrophobic side chain producing the same selective loss-of-function effect as Y32A (Figure S2).

## Discussion

The main goals of combining site-directed mutagenesis, electrophysiological assays, and modelling studies were to identify residues in the β1 subunit that play roles in VGSC current modulation and acquire hints about the location of the interface between the subunits. Voltage-gated ion channels are highly flexible complexes that undergo conformational changes to transition between resting, activated, inactivated, and closed states to respond to membrane depolarization [31]. Therefore, we considered the possibility of multiple binding modes between the α and β1 subunits.

In this study, the selective effects of the β1 point mutants suggested the existence of multiple functional binding modes that affected channel processes differently. Our interpretation is consistent with the state model for reversible protein-protein associations, in which conformational rearrangements involving side chain movements optimize binding [32].

A number of electrophysiological studies, including single channel recordings and simulations using Eyring reaction rate theory models has supported the assumption that the β1 subunit modulates VGSC currents by shifting the fraction of channels in fast and slow gating modes [6], [33], [34]. This implies that α subunits alone are able to inactivate and recover in a fast gating mode, but the probability is low because the process is thermodynamically unfavourable [34]. Consequently, the binding of β1 to the α subunit may reduce the energy barrier and increase the probability of fast mode gating. Our experimental findings and computed predictions are consistent with these assumptions, they also suggest that the mechanisms by which β1 accelerates the kinetics of sodium channels and modulates voltage dependency are independent.

Here, the electrophysiological results revealed that R89A decreased the β1-induced acceleration of inactivation and recovery from inactivation without affecting the voltage dependence or the availability after repetitive depolarisations. Conversely, Y32A and G33M, point mutants in the first loop of the β1 extracellular domain, selectively affected the voltage dependence of inactivation without affecting the kinetics. Qu *et al.* discovered similar site-specific effects on β1-induced modulation of sodium channel α subunits [9]. Their results with chimeric Nav1.2/Nav1.5 channels showed that the substitution of a single extracellular loop in domain IV decreased the ability of β1 to modulate the kinetics of inactivation and recovery without major effects on the voltage dependence [9].

The extracellular domain of the VGSC β1subunit and the extracellular vestibule of the pore-forming α subunit were predicted to be highly acidic. Our electrostatic calculations indicated a net charge of −9 (under CHARMM) and −8.46 (under AMBER) for β1 and a polar area of 3406.3 Å^2^ (over 142 resides in the extracellular domain). We substituted arginine in position 89 to eliminate the positive charge on the β1 extracellular domain (Figure 5 A).

Based on *in silico* studies, we hypothesized that R89 acted within its side chain to form a solvent exposed salt bridge, which could be broken as it approached the external vestibule of the α subunit. After depolarization, R89 may directly bind the α subunit. The simulated side-flipping contribution from the intracatenar to intercatenar protein-protein association supports the findings indicating that a shift in the fraction of VGSCs that undergo rapid inactivation and recovery could occur without modifying the midpoint voltage of inactivation.

In contrast, the C43A mutant produced general loss-of-function effects, possibly due to an increase in translational entropy caused by the loss of the theoretical disulphide bond with C21. This may allow the N-terminal loop in which this cysteine is located more flexibility (Figure 5 B). Based on the comparisons with crystal structures, we argue that this structural change could not catastrophically affect folding. Indeed, none of the Ig-like proteins form disulphide bonds at this site although they conserved antiparallel β-sheet motifs (Figure 1 B). However, we cannot rule out the possibility that the cytoskeletal trafficking or the membrane insertion of the mutants was diminished, therefore current amplitudes between groups cannot be compared. Other studies have obtained similar results on the VGSC β3 subunit. The C24A point mutant in β3, which corresponded to C43A in β1, shifted the voltage of activation and modified the half voltage of inactivation of the Nav1.5 channel [35]. In another series of studies, C24A prevented homophilic binding as shown by immunoprecipitation assays in HEK293 cells. The interpretation was that the disulphide bond formed by C24 does not directly participate in the binding site for dimerization but that it contributes to orienting the interacting residues [29].

The reductions in the fractions of mutant channels recovered after the first millisecond were 95% for C43A and 90% for R89A, and the changes remained significant after a Δt of 10 ms for R89A and after a Δt of 100 ms for C43A (figure 1 A). These effects seem consequential according to the literature. Importantly, the evidence for the principles ruling β1 association to the α subunit have not advanced in over 14 years [5], [10]. These studies indicate that an acid triad domain (E23, D25, E27) in the extracellular domain of β1 located between C43 and Y32 plays a key role. Expression of the β1 triple mutant E23Q D25Q E27Q with the Nav 1.2 channel reduced the fraction of channels that underwent rapid inactivation by 22% compared to WT Nav1.2+β1 [10]. Point mutations and larger sequence manipulations caused loss-of-function effects with comparable magnitudes to changes elicited by C43A and R89A [10].

The biophysical response to the R89A substitution seemed relevant compared to R85C or R85H, two mutations on the β1 subunit associated with generalized epilepsy with febrile seizures plus (GEFS+) [36]. Xu *et al.* (2007) found that R85C but not R85H affected current inactivation of the Nav1.2 channels [36]. After β1 WT model refinement, we found that R85 had the potential to form electrostatic interactions with nearby acidic residues, similar to R89. The reported discrepancy was computationally simulated. The results indicated that substituting histidine for arginine could rearrange the non-covalent bond network and that cysteine replacement disrupts it.

In addition, our model predicts that E87 forms a salt bridge with R89 and thus may be of functional relevance. Indeed the E87Q mutation is associated with Brugada syndrome, a channelopathy identified in a Turkish family [37]. The C121W mutation was also associated with GEFS+, and biochemical studies have indicated that C121 forms an extracellular intramolecular disulphide bridge that was disrupted by this mutation [38]. Our β1 model suggested that C40 would be its most probable binding partner (Figure 1A).

The deceleration of recovery and the increase in rundown on high frequency stimulation may be important factors that restrain hyper-excitability in pain conduction and conditions such as epilepsy [11]. Interestingly, R89A delayed the recovery from inactivation without increasing the rundown, and C43A disrupted both β1-induced effects.

Our results were consistent with the Monod-Wyman-Changeux model in that: (I) mutations can shift the spontaneous allosteric equilibrium between relaxed and tense conformational states of the complex (in this case fast versus slow gating and voltage sensitive to voltage hypersensitive states), and (II) in a system of interacting protein subunits (or protomers) within a membrane lattice, various classes of responses to specific regulatory signals can exist, from graded to all-or-none phase transitions, depending on the isomerisation and the free energy of the interaction between protomers in large and periodic protein assemblies [39].

In this study, we suggest the existence of 2 or more discrete α-β1 binding states, one that controls rapid gating and another that dictates voltage sensing modulation. Taken together, these data may enhance the understanding of pathologies associated with the interactions of α and β1 in VGSCs and enable the design of molecular compounds that selectively interfere with a particular electrophysiological process.

## Supporting Information

Figure S1 **Multiple sequence alignment of β1 sequences.** Vertebrate β1 sequences in gray (*Danio rerio, Sternopygus macrurus, Osmerus mordax, Takifugu rubripes, Homo sapiens, Macaca mulatta, Canis lupus familiaris, Mus musculus, Oryctolagus cuniculus*) aligned with 3 related proteins including the rat β2 subunit, the frog β3 subunit, and the rat myelin protein P0 (lines 10–12 in brown). Highly conserved residues were highlighted in cyan. The acid triad domain (ExDxD) was implicated in the modulation of the VGSC brain isoform (first panel in red). Substitutionss of either Cys or His for Arg85 (second panel in red) were related to familial cases of GEFS+. Regions highlighted in yellow and green form β strands and α helixes, respectively. Consensus secondary structure predictions for the β sequence (performed by Jpred) were presented on line 14.
(TIF)

Figure S2 **Effects of C43A and R89A β1 mutants on the electrostatic surface potential (ESP) of the extracellular domain.** ESP represented by Coulombic colouring within a radius of 5 Å from residue 32 (in the left panel). The substitution of tyrosine (Y32A) increased the polarity by destabilizing a networked coordination of intermolecular H-bonds. This was caused by the loss of the hydroxyl group, which shared a hydrogen with Asp148. The hydrophobicity was reduced by the loss the aromatic phenyl ring. The ESP within a radius of 5 Å from position 33 (in the right panel) showed the introduction of a long hydrophobic side chain in the G33M mutant. Calculations were made under the AMBER-Gasteiger force field in Chimera 1.3.5 after energy minimization under AMMP in VEGA ZZ 2.3.2.
(TIF)

## References

[1] Catterall WA (2000) From ionic currents to molecular mechanism: the structure and function of voltage-gated sodium channels. Neuron 26: 13–25. 10798388 10.1016/s0896-6273(00)81133-2

[2] Brackenbury WJ, Isom LL (2008) Voltage-gated Na+ channels: Potential for β subunits as therapeutic targets. Expert Opin Ther Targets 12: 1191–1203. 18694383 10.1517/14728222.12.9.1191 PMC3376311

[3] Catterall WA, Goldin AL, Waxman SG (2005) International Union of Pharmacology XLVII Nomenclature and structure-function relationships of voltage-gated sodium channels. Pharmacol. Rev 57: 397–409. 10.1124/pr.57.4.4 16382098

[4] Hille B (2001) Ion channels of excitable membranes. Sunderland, MA: Sinauer Associates Inc Press. 251 p.

[5] Brackenbury WJ, Isom LL (2011) Na Channel β Subunits: Overachievers of the Ion Channel Family. Front Pharmacol 28: 2–53. 10.3389/fphar.2011.00053 PMC3181431 22007171

[6] Patton DE, Isom LL, Catterall WA, Goldin AL (1994) The Adult Rat Brain Subunit Modifies Activation and Inactivation Gating of Multiple Sodium Channels. J Biol Chem 269: 17649–17655. 8021275

[7] Chen C, Cannon SC (1995) Modulation of Na+ channel inactivation by the b1 subunit: a deletion analysis. Pflügers Arch-Eur Physiol 43l: 186–195. 10.1007/BF00410190 9026778

[8] Makita N, Bennett PB, George AL Jr (1996) Molecular determinants of beta 1 subunit-induced gating modulation in voltage-dependent Na+ channels. J Neurosci 16(22): 7117–7127. 8929421 10.1523/JNEUROSCI.16-22-07117.1996 PMC6578941

[9] Qu Y, Rogers JC, Chen SF, McCormick KA, Scheuer T, et al (1999) Functional roles of the extracellular segments of the sodium channel alpha subunit in voltage-dependent gating and modulation by beta1 subunits. J Biol Chem 274(46): 32647–32654. 10551819 10.1074/jbc.274.46.32647

[10] McCormick KA, Srinivasan J, White K, Scheuer T, Catterall WA (1999) The Extracellular Domain of the β1 Subunit Is Both Necessary and Sufficient for β1-like Modulation of Sodium Channel Gating. J Biol Chem 274: 32638–32646. 10551818 10.1074/jbc.274.46.32638

[11] Meadows LS, Malhotra J, Loukas A, Thyagarajan V, Kazen-Gillespie KA, et al (2002) Functional and Biochemical Analysis of a Sodium Channel beta 1 Subunit Mutation Responsible for Generalized Epilepsy with Febrile Seizures Plus Type 1. J Neurosci 22: 10699–10709. 12486163 10.1523/JNEUROSCI.22-24-10699.2002 PMC6758463

[12] Patino GA, Isom LL (2010) Electrophysiology and beyond: multiple roles of Na+ channel β subunits in development and disease. Neurosci Lett 486(2): 53–59. 20600605 10.1016/j.neulet.2010.06.050 PMC2964441

[13] Zimmer T, Benndorf K (2002) The human heart and rat brain IIA Na+ channels interact with different molecular regions of the β1 subunit. J Gen Physiol 120: 887–895. 12451056 10.1085/jgp.20028703 PMC2229568

[14] Nguyen HM, Miyazaki H, Hoshi N, Smith BJ, Nukina N, et al (2012) Modulation of voltage-gated K+ channels by the sodium channel β1 subunit. Proc Natl Acad Sci U S A 109(45): 18577–18582. 23090990 10.1073/pnas.1209142109 PMC3494885

[15] Valencia A, Pazos F (2006) Prediction of protein-protein interactions from evolutionary information. Methods Biochem Anal 44: 411–426. 12647397

[16] Ofran Y, Rost B (2007) Protein-protein interaction hotspots carved into sequences. PLoS Comput Biol 3(7): e119. 17630824 10.1371/journal.pcbi.0030119 PMC1914369

[17] Orta-Salazar G (2004) Mecanismo de acción de la primaquina sobre el canal de Na+ Nav1.5 y Nav1.4 expresado en ovocitos de la rana Xenopus laevis. PhD Thesis.

[18] Moran O, Nizzari M, Conti F (2000) Endogenous expression of the beta1A sodium channel subunit in HEK-293 cells. FEBS Lett 473(2): 132–134. 10812059 10.1016/s0014-5793(00)01518-0

[19] Zhang Y (2008) I-TASSER server for protein 3D structure prediction. BMC Bioinformatics 9: 40–48. 18215316 10.1186/1471-2105-9-40 PMC2245901

[20] Combet C, Jambon M, Deleage G, Geourjon C (2002) Geno3D: automatic comparative molecular modelling of protein. Bioinformatics 18: 213–214. 11836238 10.1093/bioinformatics/18.1.213

[21] Nielsen M, Lundegaard C, Lund O, Petersen TN (2010) CPHmodels-3.0 Remote homology modeling using structure-guided sequence profiles. Nucleic Acids Res l, 38 (Web Server issue): W576–81. 10.1093/nar/gkq535 PMC2896139 20542909

[22] Pedretti A, Villa L, Vistoli G (2004) VEGA–an open platform to develop chemo-bio-informatics applications, using plug-in architecture and script programming. J Comput Aided Mol Des 18: 167–173. 15368917 10.1023/b:jcam.0000035186.90683.f2

[23] Meng EC, Pettersen EF, Couch GS, Huang CC, Ferrin TE (2006) Tools for integrated sequence-structure analysis with UCSF Chimera. BMC Bioinformatics 7: 339–349. 16836757 10.1186/1471-2105-7-339 PMC1570152

[24] Willard L, Ranjan A, Zhang H, Monzavi H, Boyko RF, et al (2003) VADAR: a web server for quantitative evaluation of protein structure quality. Nucleic Acids Res 31: 3316–3319. 12824316 10.1093/nar/gkg565 PMC168972

[25] Snider C, Jayasinghe S, Hristova K, White SH (2009) MPEx: A tool for exploring membrane proteins. Protein Sci 18: 2624–2628. 19785006 10.1002/pro.256 PMC2821280

[26] Cole C, Barber JD, Barton GJ (2008) The Jpred 3 secondary structure prediction server. Nucleic Acid Res: 36(Web Server issue): W197–201. 10.1093/nar/gkn238 PMC2447793 18463136

[27] Edgar RC (2004) MUSCLE: multiple sequence alignment with high accuracy and high throughput Nucleic Acids Res. 32(5): 1792–1797. 10.1093/nar/gkh340 PMC390337 15034147

[28] Smith TF, Waterman MS (1981) Identification of common molecular subsequences. J Mol Biol 147(1): 195–197. 7265238 10.1016/0022-2836(81)90087-5

[29] Yereddi NR, Cusdin FS, Namadurai S, Packman LC, Monie TP, et al (2013) The immunoglobulin domain of the sodium channel β3 subunit contains a surface-localized disulfide bond that is required for homophilic binding. FASEB J 27(2): 568–580. 23118027 10.1096/fj.12-209445 PMC3583845

[30] Scior T, Wahab HA (2007) Structure prediction of proteins with very low homology. In: Drug Design Research Perspectives. New York, NY: Nova Science Publishers. 675–708 pp.

[31] O’Reilly AO, Charalambous K, Nurani G, Powl AM, Wallace BA (2008) G219S mutagenesis as a means of stabilizing conformational flexibility in the bacterial sodium channel NaChBac. Mol Membr Biol 25(8): 670–676. 18991143 10.1080/09687680802508754

[32] Xu D, Tsai CJ, Nussinov R (1997) Hydrogen bonds and salt bridges across protein–protein interfaces. Protein Eng 10(9): 999–1012. 9464564 10.1093/protein/10.9.999

[33] Isom LL, De Jongh KS, Patton DE, Reber BF, Offord J, et al (1992) Primary structure and functional expression of the beta 1 subunit of the rat brain sodium channel. Science 256(5058): 839–842. 1375395 10.1126/science.1375395

[34] Bennett PB Jr, Makita N, George AL Jr (1993) A molecular basis for gating mode transitions in human skeletal muscle Na+ channels. FEBS Lett 326(1–3): 21–24. 8391996 10.1016/0014-5793(93)81752-l

[35] Yu EJ, Ko SH, Lenkowski PW, Pance A, Patel MK, et al (2005) Distinct domains of the sodium channel beta3-subunit modulate channel-gating kinetics and subcellular location. Biochem J 392(Pt 3): 519–526. 10.1042/BJ20050518 PMC1316291 16080781

[36] Xu R, Thomas EA, Gazina EV, Richards KL, Quick M, et al (2007) Generalized epilepsy with febrile seizures plus-associated sodium channel beta subunit mutations severely reduce beta subunit-mediated modulation of sodium channel function. Neuroscience 148(1): 164–174. 17629415 10.1016/j.neuroscience.2007.05.038

[37] Watanabe H, Koopmann TT, Le Scouarnec S, Yang T, Ingram CR, et al (2008) Sodium channel β1 subunit mutations associated with Brugada syndrome and cardiac conduction disease in humans. J Clin Invest 118(6): 2260–2268. 18464934 10.1172/JCI33891 PMC2373423

[38] Barbieri R, Baroni D, Moran O (2012) Identification of an intra-molecular disulfide bond in the sodium channel β1-subunit. Biochem Biophys Res Commun 420(2): 364–367. 22425777 10.1016/j.bbrc.2012.02.163

[39] Changeux JP (2012) Allostery and the Monod-Wyman-Changeux model after 50 years. Annu Rev Biophys 41: 103–133. 22224598 10.1146/annurev-biophys-050511-102222

