# Supplementary figures and images for: Identification of Navβ1 Residues Involved in the Modulation of the Sodium Channel Nav1.4
Source: PLoS One. 2013 Dec 16;8(12):e81995. doi: 10.1371/journal.pone.0081995 (PMC3864903; doi:10.1371/journal.pone.0081995)

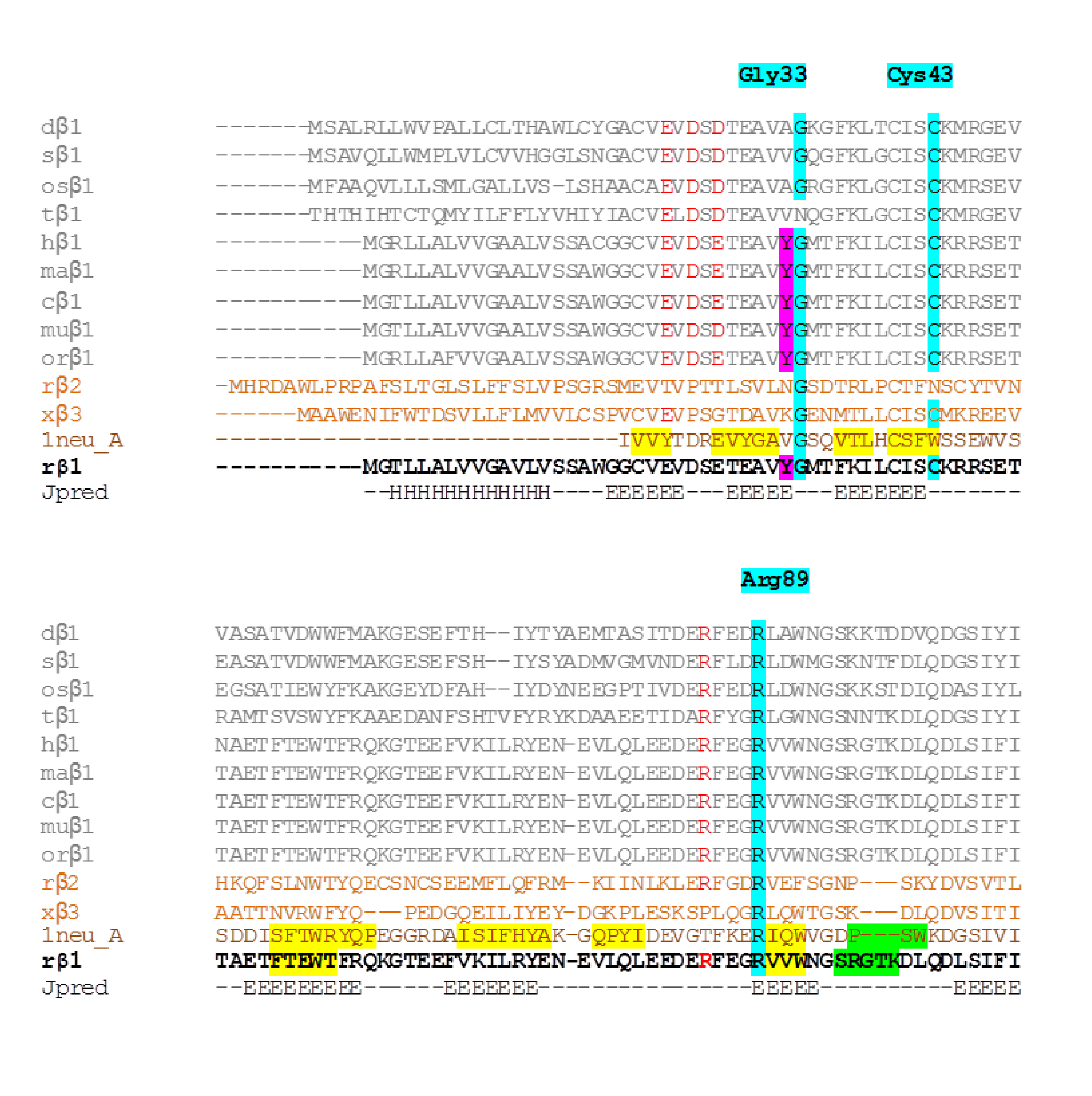

Supplement: Figure S1 — Multiple sequence alignment of β1 sequences. Vertebrate β1 sequences in gray (Danio rerio, Sternopygus macrurus, Osmerus mordax, Takifugu rubripes, Homo sapiens, Macaca mulatta, Canis lupus familiaris, Mus musculus, Oryctolagus cuniculus) aligned with 3 related proteins including the rat β2 subunit, the frog β3 subunit, and the rat myelin protein P0 (lines 10–12 in brown). Highly conserved residues were highlighted in cyan. The acid triad domain (ExDxD) was implicated in the modulation of the VGSC brain isoform (first panel in red). Substitutionss of either Cys or His for Arg85 (second panel in red) were related to familial cases of GEFS+. Regions highlighted in yellow and green form β strands and α helixes, respectively. Consensus secondary structure predictions for the β sequence (performed by Jpred) were presented on line 14. (TIF) [file pone.0081995.s001.tif]

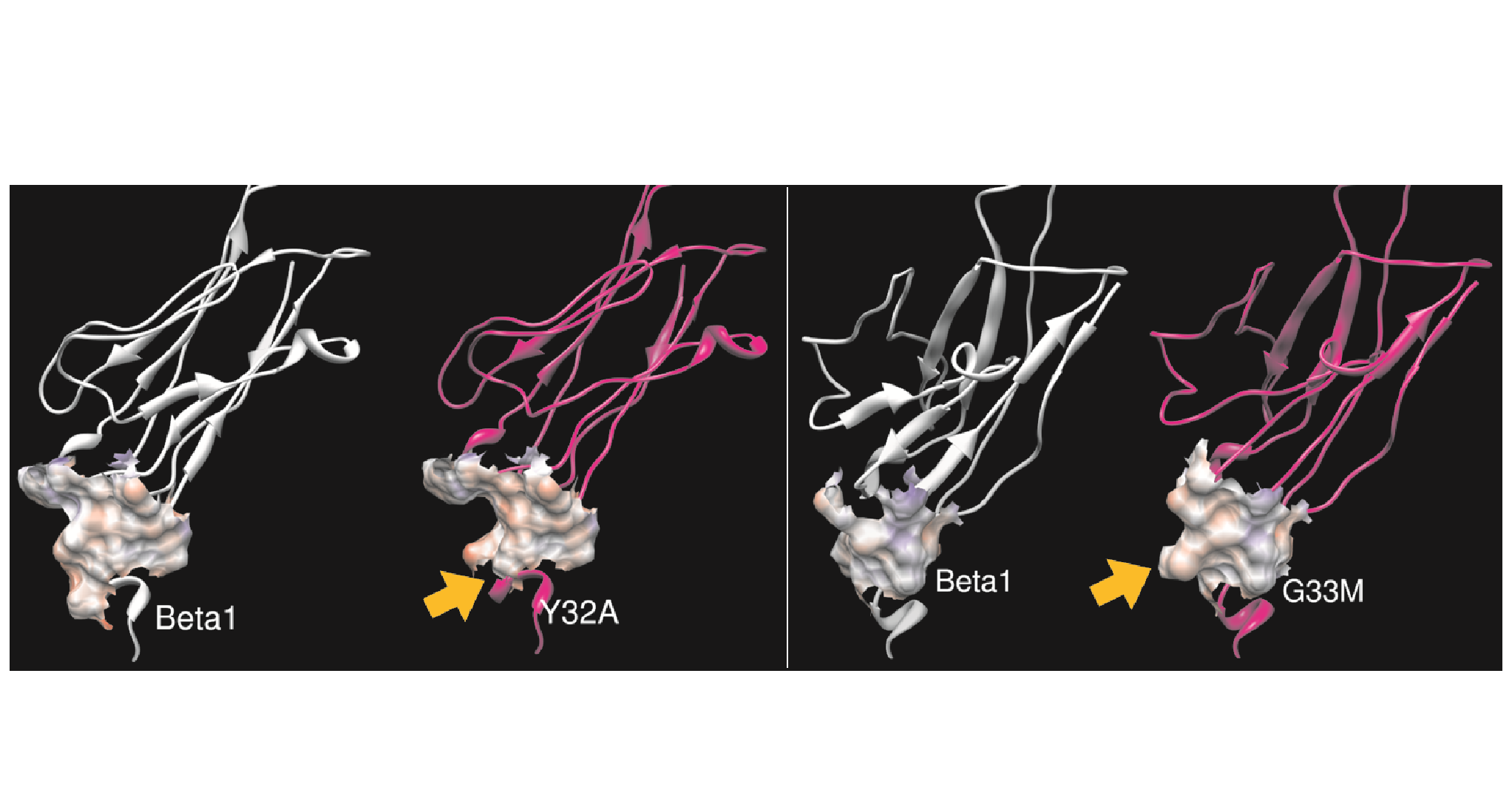

Supplement: Figure S2 — Effects of C43A and R89A β1 mutants on the electrostatic surface potential (ESP) of the extracellular domain. ESP represented by Coulombic colouring within a radius of 5 Å from residue 32 (in the left panel). The substitution of tyrosine (Y32A) increased the polarity by destabilizing a networked coordination of intermolecular H-bonds. This was caused by the loss of the hydroxyl group, which shared a hydrogen with Asp148. The hydrophobicity was reduced by the loss the aromatic phenyl ring. The ESP within a radius of 5 Å from position 33 (in the right panel) showed the introduction of a long hydrophobic side chain in the G33M mutant. Calculations were made under the AMBER-Gasteiger force field in Chimera 1.3.5 after energy minimization under AMMP in VEGA ZZ 2.3.2. (TIF) [file pone.0081995.s002.tif]
